# Manganese (II) Complex of 1,4,7-Triazacyclononane-1,4,7-Triacetic Acid (NOTA) as a Hepatobiliary MRI Contrast Agent

**DOI:** 10.3390/ph16040602

**Published:** 2023-04-17

**Authors:** Md. Kamrul Islam, Ah-Rum Baek, Byeong-Woo Yang, Soyeon Kim, Dong Wook Hwang, Sung-Wook Nam, Gang-Ho Lee, Yongmin Chang

**Affiliations:** 1 Institute of Biomedical Engineering Research, Kyungpook National University, Daegu 41405, Republic of Korea; 2 Department of Medical and Biological Engineering, School of Medicine, Kyungpook National University, Daegu 41944, Republic of Korea; 3 Division of RI-Convergence Research, Korea Institute of Radiological and Medical Sciences, Seoul 01812, Republic of Korea; 4 Department of Molecular Medicine, School of Medicine, Kyungpook National University, Daegu 41944, Republic of Korea; 5 Department of Chemistry, Kyungpook National University, 80, Daehak-ro, Buk-gu, Daegu 41566, Republic of Korea; 6 Department of Radiology, Kyungpook National University Hospital, Daegu 41944, Republic of Korea

**Keywords:** magnetic resonance imaging, manganese, contrast agent, naphthalene, liver imaging

## Abstract

Magnetic resonance imaging (MRI) is increasingly used to diagnose focal and diffuse liver disorders. Despite their enhanced efficacy, liver-targeted gadolinium-based contrast agents (GBCAs) raise safety concerns owing to the release of toxic Gd^3+^ ions. A π-conjugated macrocyclic chelate, **Mn-NOTA-NP**, was designed and synthesized as a non-gadolinium alternative for liver-specific MRI. **Mn-NOTA-NP** exhibits an *r*_1_ relaxivity of 3.57 mM^−1^ s^−1^ in water and 9.01 mM^−1^ s^−1^ in saline containing human serum albumin at 3 T, which is significantly greater than the clinically utilized Mn^2+^-based hepatobiliary drug, Mn-DPDP (1.50 mM^−1^ s^−1^), and comparable with that of GBCAs. Furthermore, the in vivo biodistribution and MRI enhancement patterns of **Mn-NOTA-NP** were similar to those of the Gd^3+^-based hepatobiliary agent, Gd-DTPA-EOB. Additionally, a 0.05 mmol/kg dose of **Mn-NOTA-NP** facilitated high-sensitivity tumor detection with tumor signal enhancement in a liver tumor model. Ligand-docking simulations further indicated that **Mn-NOTA-NP** differed from other hepatobiliary agents in their interactions with several transporter systems. Collectively, we demonstrated that **Mn-NOTA-NP** could be a new liver-specific MRI contrast agent.

## 1. Introduction

Magnetic resonance imaging (MRI) is a non-invasive technique that generates high-resolution images of the soft tissues of living organisms and has become an increasingly popular diagnostic tool in the last few decades [1,2,3]. Notably, high-resolution imaging techniques have considerably facilitated the detection and treatment of small liver lesions and other hepatic disorders [4,5]. Only two gadolinium-based contrast agents (GBCAs) are currently available for clinical liver MRI: gadoxetic acid (Gd-DTPA-EOB) and gadobenic acid (Gd-BOPTA). These agents use hydrophobic ligands to accumulate in hepatocytes via organic anion-transporting polypeptides (OATPs) [6]. Despite their high *T*_1_-weighted contrast, long-term gadolinium (Gd^3+^) retention in the brain and bones, as well as the development of nephrogenic systemic fibrosis (NSF) in patients with impaired kidney function, has raised serious safety concerns regarding GBCAs [7,8]. Owing to these issues and the lack of non-gadolinium contrast agents (CAs) in clinical settings, feasible alternatives are required.

Multiple approaches have been employed to address the concerns regarding GBCAs, such as using Mn-based CAs, superparamagnetic iron oxide nanoparticles, chemical exchange saturation transfer imaging, X-nuclei MRI, and hyperpolarized imaging [9]. Among them, Mn-based CAs have kinetics and a biodistribution comparable with that of GBCAs in living organisms. They do not require unique pulse sequences, additional post-processing, or special equipment. Biologically active manganese (II) (Mn^2+^) chelates have become the focus of MRI CA studies [10,11,12]. Notably, complexes of biocompatible paramagnetic Mn^2+^ ions with five unpaired electrons [3d^5^] have attracted attention owing to their physical features, including delayed longitudinal electronic relaxation, shorter metal–water proton effect distances, and labile water exchange kinetics [13,14]. Additionally, homeostasis and transport in biological systems ensure effective Mn elimination [15]. Mn was the first element employed as an MRI CA in vivo, and mangafodipir trisodium (Mn-DPDP) is the only FDA-approved Mn-based chelate for liver imaging [16,17]. However, the clinical use of Mn-DPDP has been discontinued owing to its low stability. Despite the report of several stable linear Mn^2+^-based chelates for use as hepatobiliary MRI CAs [18,19,20,21,22], the lack of ligand field stabilization energy in the high-spin (d^5^) electronic state has reduced the stability of the Mn^2+^ chelates [11]. Nevertheless, the increased stability of macrocyclic compounds, on the other hand, is determined by the macrocyclic effect. Therefore, macrocyclic chelates remain the most appealing option for developing highly inert Mn-based chelates.

In this study, we describe the synthesis, characterization, and in vivo biological evaluation of an amphiphilic macrocyclic Mn^2+^ chelate (**Mn-NOTA-NP**) as a biocompatible liver-specific MRI CA. The stability of **Mn-NOTA-NP** was compared with that of a linear Mn^2+^ chelate (Mn-EDTA). In addition, the lipophilic naphthalene (NP) moiety attached to the NOTA backbone by π-conjugation promotes hepatocyte targeting of the **Mn-NOTA-NP** chelate [23]. Hence, we demonstrated that this novel macrocyclic Mn^2+^ chelate (**Mn-NOTA-NP**) could be a feasible alternative to GBCAs for liver imaging.

## 2. Results and Discussion

### 2.1. Synthesis and Characterization

**Scheme 1:**
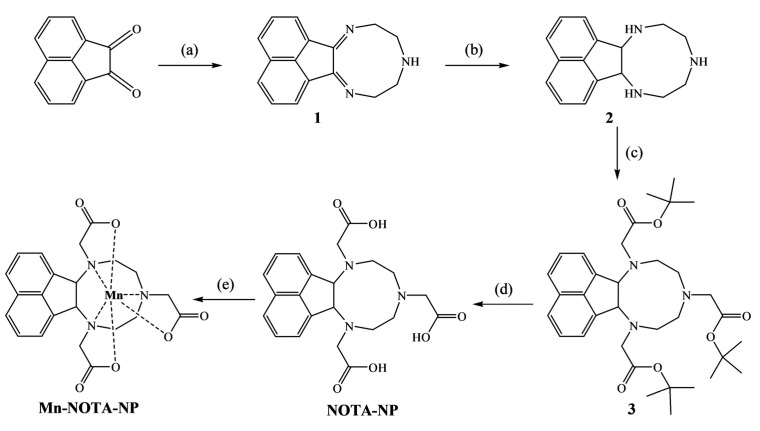
Synthesis of the **Mn-NOTA-NP** complex ^a^. ^a^ Reagents and conditions: (**a**) Diethylenetriamine, citric acid, EtOH, 3 h, room temperature (rt); (**b**) NaBH_4_, MeOH, 0 °C, overnight; (**c**) *tert*-butyl bromoacetate, K_2_CO_3_, acetonitrile, rt; (**d**) TFA, CH_2_Cl_2_, 18 h, rt; (**e**) MnCl_2_·4H_2_O, 1 M NaOH, pH = 7.0.

The **NOTA-NP** ligand was synthesized by multiple-step reactions, starting with acenaphthylene-1,2-dione, and the synthetic pathway for **Mn-NOTA-NP** is depicted in Scheme 1. First, compound **2** was synthesized according to a published procedure with slight modifications [24], and alkylation with three equivalents of *tert*-butyl-bromoacetate provided protected ligand **3**. Subsequently, the *tert*-butyl group was removed using trifluoroacetic acid (TFA), and a pure chelating ligand (**NOTA-NP**) was isolated by precipitation with ether. The Mn^2+^ complex was then synthesized by reacting the ligand with an equivalent of MnCl_2_.4H_2_O at pH 7.0, followed by the removal of inorganic impurities by reverse-phase chromatography. Finally, the pure chelate was collected as an off-white solid via lyophilization. The results of microanalysis and spectroscopic techniques, such as ^1^H NMR, ^13^C NMR, HR-FAB-MS, HR-ESI-MS, and elemental analysis, confirmed ligand and complex formation (Figures S1–S9). The purity of **Mn-NOTA-NP** was 96.05% (Figure S10).

### 2.2. Relaxivities and Human Serum Albumin (HSA)-Binding of **Mn-NOTA-NP**

The efficiency of the MRI CAs was measured in terms of their *r*_1_ and *r*_2_ relaxivities (in mM^−1^ s^−1^), which are defined as variations in the relaxation rate of the water protons. At 3 T, we determined the relaxivities of **Mn-NOTA-NP** and compared them with those of Mn-EDTA, Mn-DPDP, Gd-DTPA, Gd-DTPA-EOB, and Gd-BOPTA. **Mn-NOTA-NP** has an *r*_1_ value of 3.57 mM^−1^ s^−1^, which is 2.0-, 2.3-, and 2.4-fold higher than those of Mn-NOTA, Mn-EDTA, and Mn-DPDP, respectively (Table 1) [25,26]. NOTA, a hexadentate ligand, has three oxygen and three nitrogen donor atoms [27]. Mn-NOTA chelate is not saturated because Mn^2+^ can form seven coordinated complexes. To complete seven coordinations, a water molecule can bind in the inner sphere of the Mn chelate. In the presence of 0.67 mM HSA, the *r*_1_ relaxivity was increased to 9.01 mM^−1^ s^−1^, which is higher than that of Mn-EDTA and Gd-BOPTA, but relatively lower than Gd-DTPA-EOB. This suggests that it could be used in *T*_1_-weighted acquisitions with non-GBCAs (Table 1). The π-conjugated lipophilic naphthalene group facilitates albumin binding to **Mn-NOTA-NP** and slows the tumbling rate following macromolecular complex formation. Although Mn^2+^ (S = 5/2) has fewer unpaired electrons than Gd^3+^ (S = 7/2), the closer proximity of Mn^2+^ to the water molecules may compensate for this reduced number [28]. The *r*_2_/*r*_1_ ratio is vital for determining the type of MRI CAs. A CA with a low *r*_2_/*r*_1_ ratio (<5) has a *T*_1_-dominated contrast, whereas a CA with a high *r*_2_/*r*_1_ ratio (>8) has a *T*_2_-dominated contrast [29,30].

We also measured the binding constant (K_a_), which reflects the interaction of **Mn-NOTA-NP** with HSA (Figure S11). The binding constant K_a_ (36.6 M^−1^) for **Mn-NOTA-NP** was comparable to the clinically used liver imaging agent Gd-DTPA-EOB (27 M^−1^) [31]. Poor albumin binding increased relaxivity; however, it did not affect plasma enhancement.

**Table 1:** Relaxivities and lipophilicity of **Mn-NOTA-NP**, Mn-EDTA, Mn-DPDP, Gd-DTPA, Gd-DTPA-EOB, and Gd-BOPTA in water and human serum albumin (HSA) at 3 T (297 K).

Compound	Water			HSA (0.67 mM)			HSA Binding Constant (K_a_)	logP oct/wat
	*r*_1_ (mM^−1^ s^−1^)	*r*_2_ (mM^−1^ s^−1^)	*r*_2_/*r*_1_	*r*_1_ (mM^−1^ s^−1^)	*r*_2_ (mM^−1^ s^−1^)	*r*_2_/*r*_1_		
**Mn-NOTA-NP**	3.57 ± 0.20	18.08 ± 0.50	5.0	9.01 ± 0.50	42.83 ± 2.70	4.8	36.6 M^−1^	−0.93
**Mn-EDTA**	1.79 ± 0.05	3.85 ± 0.05	2.2	2.93 ± 0.30	5.54 ± 0.10	1.9	-	−2.72
**Mn-DPDP ^b^**	1.50 ± 0.40	2.30 ± 0.60	1.5	-	-	-	-	−3.07
**Gd-DTPA**	4.12 ± 0.10	5.20 ± 0.20	1.3	-	-	-	-	−3.16
**Gd-DTPA-EOB ^b^**	4.30 ± 0.60	5.50 ± 0.60	1.3	9.97 ± 1.00	10.76 ± 0.10	1.1	27 M^−1^	−2.11
**Gd-BOPTA ^b^**	4.00 ± 0.60	4.70 ± 0.60	1.2	8.27 ± 0.10	10.84 ± 0.10	1.3	-	−2.23

^b^ Data were obtained from the cited reference [32].

### 2.3. Lipophilicity of **Mn-NOTA-NP**

Most Gd^3+^ complexes are confined to extracellular spaces and cannot enter cells owing to their high hydrophilicity. Typically, the biological activity of compounds increases as their lipophilicity increases owing to their attraction to biological membranes and increased permeability, allowing for greater access to the desired location inside the body [33]. We hypothesized that conjugating a lipophilic moiety to a cell-impermeable metal complex would yield an amphiphilic combination that could pass through cell membranes. The clinically applied liver imaging agent, Gd-DTPA-EOB, also comprises a hydrophilic Gd-DTPA derivative coupled with a lipophilic ethoxybenzyl (EOB) moiety. Gale et al. reported that the log P values of a series of small-molecule Mn (II) complexes are closely correlated with the blood clearance rate and liver accumulation [22]. The lipophilicity of **Mn-NOTA-NP** was estimated using octanol-water partitioning coefficient (log P) measurements. **Mn-NOTA-NP** has log *p* values of −0.93, which are 61 and 138 times greater than those of Mn-EDTA (−2.72) and Mn-DPDP (−3.17), respectively (Table 1). Furthermore, the log P value of **Mn-NOTA-NP** was 15–20 times higher than the log P values of the clinically used Gd-based agents, Gd-DTPA-EOB (−2.11) and Gd-BOPTA (−2.23) (Table 1).

### 2.4. Kinetic Inertness and pH Stability of **Mn-NOTA-NP**

**Figure 1:**
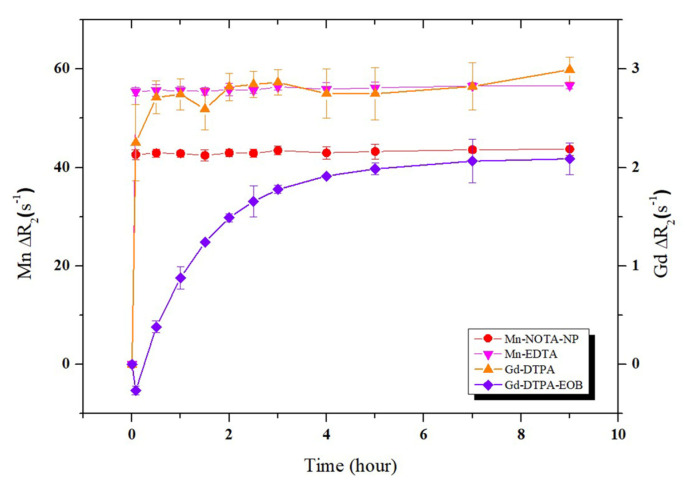
Transmetalation kinetics of 1 mM **Mn-NOTA-NP** (●), Mn-EDTA (▼), Gd-DTPA (▲), and Gd-DTPA-EOB (■) over 10 mM Zn^2+^ plotted by ΔR_2_ as a function of time at 3 T and 293 K. Data are presented as *n* = 4 and mean ± SD (standard deviation).

The kinetic inertness of metal complexes is more crucial than their thermodynamic stability for in vivo applications [14,34]. Thus, the transmetalation process between **Mn-NOTA-NP** and Zn^2+^ ions was examined using a previously published method [35]. As zinc (Zn^2+^) is the second most abundant trace metal in the human body, it can disrupt more Mn^2+^ ions than trace metals, such as Cu^2+^ and Ca^2+^. Furthermore, as the transmetalation process occurs more frequently, the chelation between the ligand and metal becomes increasingly unstable. In the current study, the kinetic inertness of **Mn-NOTA-NP** was represented by the transverse relaxivity change [Δ*r*_2_ (t) = *r*_2_ (t) − *r*_2_ (0)] as a function of time. For comparison, Mn-EDTA, Gd-DTPA, and Gd-DTPA-EOB were also assessed (Figure 1). To demonstrate this, the complexes were subjected to a tenfold excess of Zn^2+^, and the Δ*r*_2_ (t) values of the Mn^2+^ and Gd^3+^ complexes were compared. As shown in Figure 1, the ∆*r*_2_ (t) values of Mn-EDTA and Gd-DTPA rapidly increased and saturated at high ∆*r*_2_ values. In contrast, **Mn-NOTA-NP** began with a quickly increasing Δ*r*_2_ (t) value, but shortly reached saturation at significantly lower Δ*r*_2_ values and maintained its relaxation rate, implying that transmetalation occurred much less frequently after that point. These data indicate that **Mn-NOTA-NP** is more resistant to Zn^2+^ transmetalation than Mn-EDTA and Gd-DTPA, as evidenced by its rapid dissociation. An earlier manganese-based liver imaging agent, mangafodipir (Mn-DPDP), was licensed for liver imaging, but this form of chelated Mn acted as a prodrug and released free Mn^2+^ upon administration [36]. Moreover, the Δ*r*_2_ (t) pattern of Gd-DTPA-EOB increased over time and reached saturation at relatively low *r*_2_ values, although after 4 h, the Δ*r*_2_ (t) pattern of **Mn-NOTA-NP** was comparable to that of Gd-DTPA-EOB. This result suggests that **Mn-NOTA-NP** is more susceptible to Zn^2+^ transmetalation than Gd-DTPA-EOB.

Furthermore, the stability of **Mn-NOTA-NP** was assessed over time by measuring the *r*_2_ relaxivity in phosphate-buffered solutions (PBS) with pH values ranging from 1.0 to 10.0 (Figure S12). The *r*_2_ values remained steady over a wide pH range (up to 10.0); however, at pH 7, the *r*_2_ relaxivity decreased significantly compared to its initial values. These observations are to be anticipated when evoking the potential displacement of the inner-sphere water molecule by the phosphate anion of PBS [37]. Altering the complex pH from basic to acidic, i.e., from pH 4 to lower, increased the *r*_2_ values. Complex dissociation may be attributed to the increased relaxivity at a low pH, which is in line with an earlier study [25]. Thus, we believe that **Mn-NOTA-NP** is suitable for in vivo investigations and may be an appropriate substitute for GBCAs, particularly in populations with a compromised renal function where gadolinium exposure is a concern.

### 2.5. Protein–Ligand Docking Simulations of **Mn-NOTA-NP**

The molecular docking approach replicates the atomic interaction between a small molecule and a protein, allowing us to quantitatively investigate small-molecule behavior at target protein binding sites and elucidate critical biological processes [38]. Gd-DTPA-EOB is taken up by hepatocytes through OATPs (mostly OATP1B1 and OATP1B3) and discharged into bile canaliculi through multidrug resistance-associated protein 2 (MRP2) [39]. The activity of these transporters influences the metabolic and biliary clearance of drugs. Consequently, they can affect pharmacological efficacy or toxicity by affecting plasma concentration [40]. We postulated that OATPs could mediate the hepatic uptake of **Mn-NOTA-NP** because of their similar biodistribution pattern to Gd-DTPA-EOB [41]. The expression and localization of OATP1B1 and/or -1B3 and MRP2 in tumors may influence the degree of hepatocyte-selective enhancement. Therefore, AutoDock Vina was used to predict the protein–ligand docking simulations.

**Figure 2:**
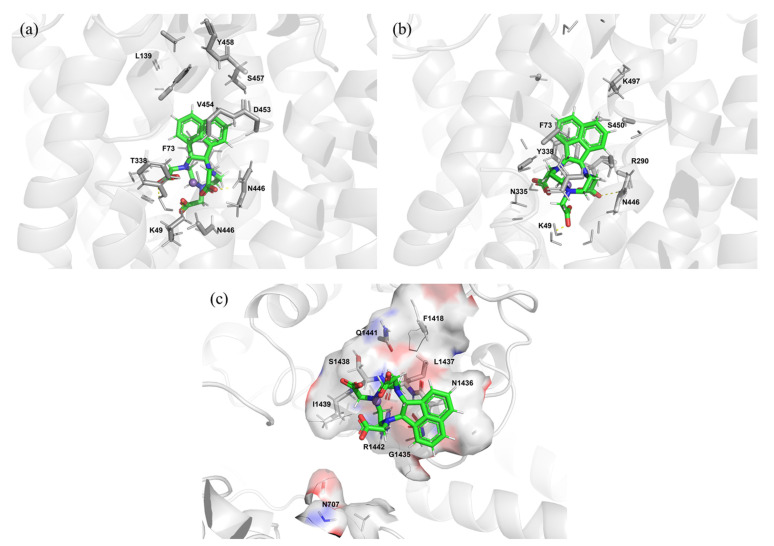
Schematic representations of variable interactions between **Mn-NOTA-NP** and (**a**) OATP1B1, (**b**) OATP1B3, and (**c**) MRP2. The yellow line represents hydrogen bonds.

The interactions between various transporter systems and **Mn-NOTA-NP** are displayed in Figure 2. **Mn-NOTA-NP** establishes a network of hydrogen bonds with the residues surrounding the OATP1B1 transporter, including N446, K49, and T338 (yellow dotted line) (Figure 2a). In addition, **Mn-NOTA-NP** interacts with OATP1B3 via hydrogen bonds at N446 and K49 (Figure 2b). For comparison, computational docking investigations of the commercially available hepatobiliary drug Gd-DTPA-EOB, as well as the previously reported Mn-EDTA-BTA and Mn-EDTA-EOB, are shown in Figures S13 and S14 [19]. Mn-EDTA-BTA forms hydrogen bonds with N446 and K49 to bind to the OATP1B1 transporter, and with N446, K49, N335, R290, S450, and K497 to bind to OATP1B3. Mn-EDTA-EOB forms multiple hydrogen bonds with the OATP1B3 transporter, and K49 forms a salt bridge with the metal complex. Gd-DTPA-EOB comprises several hydrogen bonds and salt bridges that bind to OATP1B1 and OATP1B3. **Mn-NOTA-NP** binds to a U-shaped motif composed of amino acids 1435–1442 in the MRP2 transporter (Figure 2c). Mn-EDTA-BTA, Mn-EDTA-EOB, and Gd-DTPA-EOB use the U-shaped motif of amino acids 1435–1442 as the binding site and form a hydrogen bond between R1442 and the metal complex (yellow dotted line) (Figures S13 and S14).

The AutoDock Vina scoring function was used to calculate the binding free energies of these compounds (Table 2). The docking scores of **Mn-NOTA-NP** with OATP1B1 and OATP1B3 were 9.281 and 5.532 kcal/mol, respectively. Furthermore, the docking score between **Mn-NOTA-NP** and the MRP2 transporter was 4.579 kcal/mol, which was higher than that of Gd-DTPA-EOB (3.976 kcal/mol), but lower than that of Mn-EDTA-BTA (5.731 kcal/mol) and Mn-EDTA-EOB (5.149 kcal/mol). Therefore, based on the docking analysis, the best transporter system for Mn-EDTA-BTA and Mn-EDTA-EOB was OATP1B3, whereas those of Gd-DTPA-EOB were OATP1B1 and OATP1B3. The docking simulation results revealed that **Mn-NOTA-NP** differed from the other investigated compounds in its interaction with the transporters. According to this finding, OATP1B1 is a crucial transporter for **Mn-NOTA-NP** absorption. Although OATP1B1 and OATP1B3 are expressed in normal liver tissues, their expression is reduced in primary and metastatic liver cancers [42].

### 2.6. Cytotoxicity of **Mn-NOTA-NP**

**Figure 3:**
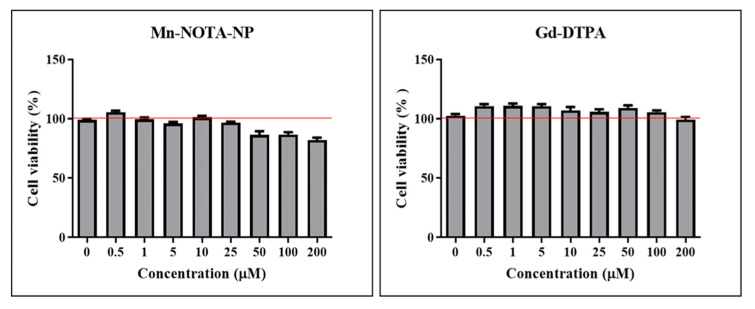
In vitro cell viability of RAW 264.7 cells with different concentrations of **Mn-NOTA-NP** and Gd-DTPA at 37 °C for 24 h. Data are represented as mean ± SEM (standard error of the mean) and *n* = 3. The red line represents the control value.

CCK-8 assays were used to assess the cytotoxic effects of **Mn-NOTA-NP** on the RAW 264.7 cell line. Cells were cultured for 24 h at 37 °C, with various doses of **Mn-NOTA-NP** and Gd-DTPA as controls (Figure 3). CCK-8 assays revealed that the cells were viable at increasing **Mn-NOTA-NP** concentrations up to 50 µM and 200 µM, which remained above 86% and 82%, respectively. Although **Mn-NOTA-NP** showed a decrease in cell viability at high concentrations, these findings indicate that **Mn-NOTA-NP** has negligible cytotoxicity at the amounts required for an optimal MRI signal.

### 2.7. In Vivo MR Images of Normal Mice Using **Mn-NOTA-NP**

**Figure 4:**
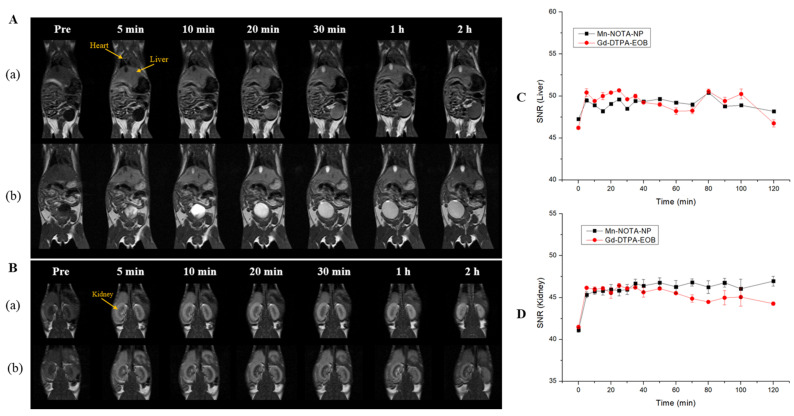
*T*_1_-weighted images of Balb/c mice recorded at 3.0 T capturing the (**A**) liver and (**B**) kidneys obtained before injection (pre) and at 5, 10, 20, and 30 min and 1 and 2 h after tail vein injection of (a) **Mn-NOTA-NP** and (b) Gd-DTPA-EOB at a dose of 0.05 and 0.025 mmol/kg, respectively. The graphs depict the signal-to-noise ratio (SNR) profiles of **Mn-NOTA-NP** (black squares) and Gd-DTPA-EOB (red circles) in the (**C**) liver and (**D**) kidneys after injection. Data are presented as mean ± SD and *n* = 3.

To test the hepatobiliary imaging capability of **Mn-NOTA-NP**, we performed in vivo MRI experiments in Balb/c mice after intravenous injection of **Mn-NOTA-NP** at 3.0 T and Gd-DTPA-EOB as a reference agent. *T*_1_-weighted coronal images of the liver (Figure 4A) and kidneys (Figure 4B) were taken before injection and at 5, 10, 20, and 30 min and 1 and 2 h after injection. Additionally, the signal strengths in the liver and kidneys were monitored, and the signal-to-noise ratio (SNR) was measured as a function of time to analyze the contrast enhancement patterns in vivo (Figure 4C,D). The most characteristic MR feature demonstrates significant and prolonged contrast enhancement in the liver after injection of (a) **Mn-NOTA-NP** or (b) Gd-DTPA-EOB at 0.05 and 0.025 mmol/kg, respectively. In vivo, MRI revealed that **Mn-NOTA-NP** has a dual pathway for excretion, which is highly similar to the clinically used Gd-DTPA-EOB. Moreover, the SNRs of **Mn-NOTA-NP** and Gd-DTPA-EOB in the liver were comparable up to 2 h after injection (Figure 4C). Notably, the injection dose of Gd-DTPA-EOB was lower than that of **Mn-NOTA-NP** in the current study because the injection dose of Gd-DTPA-EOB could not be increased owing to the risk of NSF [7,43].

Additionally, **Mn-NOTA-NP** and Gd-DTPA-EOB exhibited similar kidney signals for up to 30 min, indicating their renal excretion (Figure 4B). As demonstrated by an increase in the kidney signal after 30 min, the renal excretion of **Mn-NOTA-NP** was slightly higher than that of Gd-DTPA-EOB (Figure 4D). Overall, **Mn-NOTA-NP** with an amphiphilic negatively charged structure displayed optimal liver-specific contrast enhancement and a combination of hepatobiliary and renal clearance in this in vivo imaging study.

### 2.8. In Vivo Biodistribution of **Mn-NOTA-NP**

**Figure 5:**
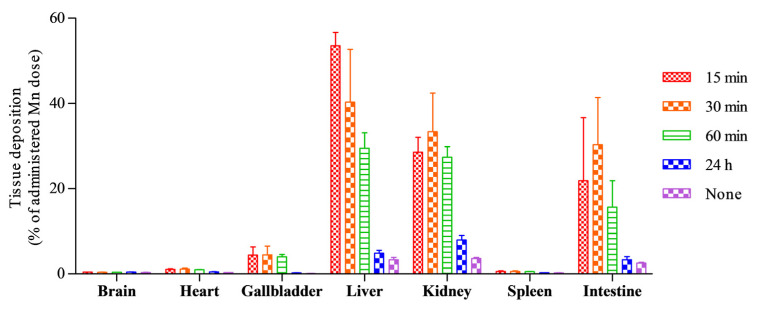
In vivo biodistribution patterns of **Mn-NOTA-NP** (0.05 mmol Mn/kg) in Balb/c mice, expressed as the percentage of Mn concentrations in each tissue (*n* = 4). Mice were sacrificed at 15 min, 30 min, 60 min, and 24 h. Untreated mice served as controls and were labeled as “None.” Data are presented as mean ± SD.

An in vivo biodistribution study was conducted to measure the concentration of Mn^2+^ ions in the brain, heart, gallbladder, liver, kidneys, spleen, and intestine using Inductively Coupled Plasma Emission Spectrometry (ICP-OES) (Figure 5). The Mn^2+^ levels were highest in the liver (approximately 54%) and intestine at 15 min, indicating that **Mn-NOTA-NP** was removed via the hepatobiliary pathway. **Mn-NOTA-NP** demonstrated a similar liver accumulation at 30 min compared to the clinically used liver-targeting agent Gd-DTPA-EOB [44,45]. Subsequently, it was rapidly removed from the liver. In addition to the liver, the kidneys also showed a high Mn accumulation owing to glomerular excretion via the renal pathway. Consequently, the in vivo biodistribution findings strongly imply that **Mn-NOTA-NP** is excreted via the hepatobiliary and renal pathways, similar to the liver-specific agent Gd-DTPA-EOB. Notably, the Mn levels were almost restored to the control levels 24 h after the **Mn-NOTA-NP** injection.

### 2.9. In Vivo MR Images of the Liver Tumor Model Using **Mn-NOTA-NP**

**Figure 6:**
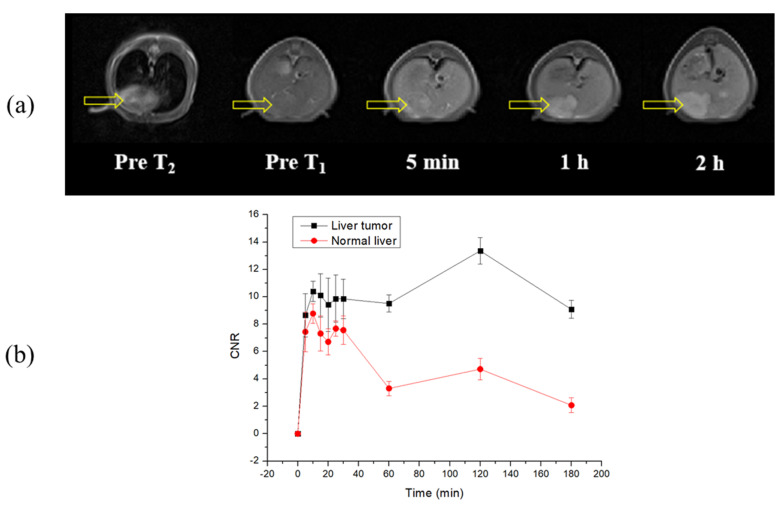
Axial *T*_2_- and *T*_1_-weighted MR images of HepG2 orthotopic mice (**a**) before injection and 5 min, 1 h, and 2 h post injection of **Mn-NOTA-NP** (0.05 mmol/kg); yellow arrows show the liver tumor lesions. (**b**) Difference of contrast-to-noise ratio (CNR) between liver tumor tissue (black squares) and normal liver parenchyma (red circles) as a function of time.

To assess the possible clinical application of the **Mn-NOTA-NP** chelate as a liver-specific CA, we performed in vivo MRI following tail vein injection using a HepG2 orthotopic mouse model of human hepatocellular carcinoma (HCC). Before administering **Mn-NOTA-NP**, *T*_1_-weighted MR images of the liver parenchyma and HCC tissues displayed indistinguishable and nonspecific iso-signal intensities. MR images captured 5 min after **Mn-NOTA-NP** injection revealed that tumor tissues could be distinguished from normal liver parenchyma because tumor tissues have a higher signal strength than normal liver parenchyma (Figure 6a). Furthermore, the HCC tissue appeared significantly hyperintense in the liver parenchyma tissue on the *T*_1_-weighted images, with high tumor signal intensity 2 h after injection of the **Mn-NOTA-NP** chelate. As a result, **Mn-NOTA-NP**-enhanced MRI would result in an elevated tumor-to-normal liver contrast ratio with high tumor detection sensitivity, allowing for the early diagnosis of HCC (Figure 6b).

Based on the molecular docking results, one possible explanation for the high tumor-to-normal liver contrast ratio after using **Mn-NOTA-NP** is the different roles of OATPs and MRP2 expression in HCC. First, **Mn-NOTA-NP**-enhanced MR images exhibited signal enhancements in both normal liver and HCC cells, suggesting that OATP1B1/B3 mediates the uptake of **Mn-NOTA-NP** from the sinusoid into the normal liver (hepatocytes) and HCC cells [6,46]. Second, HCC signal enhancement was MRP2-dependent when OATP1B1/B3 expression was maintained. As MRP2 mediates the secretion of **Mn-NOTA-NP** from tumor cells to the lumen, the decreased MRP2 expression in tumor cells results in the accumulation of **Mn-NOTA-NP** in the cytoplasm of tumor cells; thus, HCCs are hyperintense compared with the normal liver [47]. According to Ni et al., this persistent tumor enhancement could be valuable in distinguishing HCC from other liver disorders and determining HCC cellular differentiation [46,48]. However, further investigation is required to clarify the detailed mechanism of tumor enhancement by **Mn-NOTA-NP**. 

## 3. Materials and Methods

### 3.1. Reagents and Materials

The solvents were purified and dried using standard procedures. Acenaphthoquinone, diethylenetriamine, and MnCl_2_.4H_2_O were purchased from Sigma-Aldrich (St Louis, MO, USA) and used without further purification. Unless otherwise specified, all other commercial reagents were purchased from Sigma-Aldrich or Tokyo Chemical Industry Co., Ltd. (Tokyo, Japan) and used in the original forms. Deionized water was used in all experiments. The reactions were followed by thin-layer chromatography (TLC) using silica gel plates 60 F254 (Merck, Darmstadt, Germany) and UV light for visualization. ^1^H NMR (500 Hz) and ^13^C NMR (126 Hz) experiments were performed using a Bruker Advance 500 spectrometer at the Center for Instrumental Analysis, Kyungpook National University (KNU), Daegu, Korea. As an internal standard, the chemical shifts were presented as δ values of tetramethylsilane. The coupling constants are expressed in hertz. Elemental analysis was conducted at the Center for Instrumental Analysis at KNU. High-resolution fast-atom bombardment mass spectrometry (HR-FAB-MS; JMS-700, JEOL, Tokyo, Japan) and electrospray ionization time-of-flight MS (ESI-TOF-MS; SYNAPT G2, Waters, USA) were performed at the Korea Basic Science Institute. A high-pressure liquid chromatography (HPLC; LC/Forte/R, YMC, Iwata, Japan) system equipped with a Luna C18 column (250 mm × 21.2 mm, Phenomenex Inc., Torrance, CA, USA) was used for further purification. The conditions used to validate purity were as follows: eluent A, 10 mM ammonium acetate in water; eluent B, 10 mM ammonium acetate in acetonitrile; gradient, 5–40% B in 3 min, 40–80% B in 25 min, and 80–100% B in 3 min; flow rate, 12 mL/min. HPLC was performed using UV–Vis detection at 254 nm. The purity of the products was determined to be over 95% using elemental analysis and HPLC spectra. No unanticipated or exceptionally substantial safety risks were identified during the experimental procedures.

### 3.2. Synthesis and Characterization

#### 3.2.1. Synthesis of Compound **2**

The title compound was prepared using a previously reported method with slight modifications [24]. Briefly, acenaphthylene-1,2-dione (1.0 g, 5.48 mmol) was combined with diethylenetriamine (0.57 g, 5.48 mmol) in a mixture of ethanol and deionized (DI) water in a 3:1 ratio. The mixture was then agitated for 3 h with a 5-mole percent citric acid solution before being filtered. The solvent was removed, and the product was washed with acetonitrile before reusing it. Subsequently, compound **1** was dissolved in methanol, and NaBH_4_ was added in portions at 0 °C. The solvent was removed, and CH_2_Cl_2_ was used for extraction. The organic layer was dried over NaSO_4_ and then vacuumed. Using DCM: MeOH as the eluent, flash chromatography was performed to obtain a pale-yellow solid product with a yield of 78%. ^1^H NMR (500 MHz, DMSO-d_6_): δ (ppm) = 8.43–8.41 (*d*, 2H, CH, naphthalene), 8.29–8.28 (*d*, 2H, CH, naphthalene), 7.80–7.77 (*t*, 2H, CH, naphthalene), 4.35 (*s*, 2H, CH-NH), 4.12 (*t*, 2H, -CH_2_), 3.44 (*t*, 4H, -CH_2_), 2.89 (*t*, 2H, -CH_2_). ^13^C NMR (126 MHz, CDCl_3_): δ = 127.94, 125.53, 122.24, 119.40, 67.99, 30.33, 25.62. HR-FAB-MS calculated for C_16_H_19_N_3_: 254.1657 [M + H]^+^; observed: 254.1657 [M + H]^+^.

#### 3.2.2. Synthesis of Compound **3**

Compound **2** (1.0 g, 3.94 mmol) was dissolved in acetonitrile, and K_2_CO_3_ (3.26 g, 23.62 mmol) was added to the solution. Subsequently, *tert*-butyl bromoacetate (2.30 g, 11.81 mmol) was added dropwise for 30 min, and the mixture was stirred overnight at room temperature. The resulting mixture was filtered, and the solvent was removed to obtain a crude product. Compound **3** was purified by column chromatography (silica gel, hexane/EtOAc, 9:1) and collected as an oily solid; the yield was 1.41 g (61%). ^1^H NMR (500 MHz, MeOH-d_4_): δ (ppm) = 8.46–7.40 (*m*, 6H, naphthalene), 5.39 (*s*, 2H, CH), 5.21–4.84 (*m*, 1H, CH), 4.65–3.39 (*m*, 5H, CH), 3.35–3.25(*m*, 3H, CH_2_), 3.13–1.86 (*m*, 5H, CH_2_), 1.43–1.18 (*m*, 27H, CH_3_). CHN Anal. Calcd. for C_34_H_49_N_3_O_6_·K_2_CO_3_: C, 57.27; H, 6.73; N, 5.73; observed: C, 57.57; H, 6.82; N, 6.15. HR-FAB-MS calculated for C_34_H_49_N_3_O_6_: 596.3700 [M + H]^+^; observed: 596.3696 [M + H]^+^.

#### 3.2.3. Synthesis of **NOTA-NP**

Deprotection of *tert*-butyl was performed as follows: compound **3** (1.0 g, 1.68 mmol) was dissolved in a mixture of trifluoroacetic acid and CH_2_Cl_2_ at 0 °C and stirred for 18 h. The solution was evaporated under reduced pressure, and ether was added to obtain the precipitate. The product was then filtered, washed with ether several times, and vacuum-dried, and the pure product was collected as an off-white solid. The yield was 0.63 g (88%). ^1^H NMR (500 MHz, MeOH-d_4_): δ (ppm) = 7.86–7.84 (*d*, 1H, CH, naphthalene), 7.78–7.76 (*d*, 2H, CH, naphthalene), 7.58–7.53 (*m*, 3H, CH, naphthalene), 5.35–5.33 (*d*, 1H, CH), 5.21–5.19 (*d*, 1H, CH), 4.01–3.51(*m*, 6H, CH_2_), 3.40–3.25 (*m*, 4H, CH), 3.16–3.13 (*m*, 2H, CH), 2.87–2.84 (*m*, 2H, CH). ^13^C NMR (126 MHz, MeOH-d_4_): δ = 174.08, 172.60, 138.15, 131.51, 128.28, 127.70, 126.82, 125.00, 123.15, 122.03, 64.68, 64.20, 54.84, 54.73, 49.12, 44.61, 42.29. CHN Anal. Calcd. for C_22_H_25_N_3_O_6_·1.5 CF_3_COOH: C, 50.44; H, 4.50; N, 7.09; observed: C, 50.09; H, 4.43; N, 7.32. HR-FAB-MS calculated for C_22_H_25_N_3_O_6_: 428.1822 [M + H]^+^; observed: 428.1824 [M + H]^+^.

#### 3.2.4. Synthesis of **Mn-NOTA-NP**

Ligand **NOTA-NP** (0.32 g, 0.75 mmol) was dissolved in DI water, and the pH of the solution was adjusted to 7.0 by dropwise addition of 1 M NaOH. The solution of MnCl_2_.4H_2_O (0.15 g, 0.75 mmol) was added slowly, and the resulting suspension was stirred for 24 h at room temperature. The pH of the resulting mixture was readjusted to 7.0 several times. The solid was filtered and vacuum dried to obtain a crude solid product, which was further purified using preparative HPLC, and the purity was confirmed. The yield was 0.16 g (45%). CHN Anal. Calcd. for C_22_H_22_MnN_3_O_6_·3H_2_O: C, 49.54; H, 5.29; N, 7.88; observed: C, 49.37; H, 5.14; N, 8.57. HR-ESI-MS calculated for C_22_H_22_MnN_3_O_6_: 479.0889 [M]^+^; observed: 479.0889 [M]^+^.

### 3.3. Relaxivity

*T*_1_ measurements were obtained on a 3 T MRI scanner (Architect, GE Healthcare, US) using an inversion recovery method with variable inversion times (TIs). The magnetic resonance (MR) images were captured at 35 different times between 50 and 1750 ms. The signal intensity at each TI value was fitted nonlinearly to obtain the corresponding *T*_1_ relaxation time. For the *T*_2_ measurements, the Carr–Purcell–Meiboon–Gill (CPMG) pulse sequence was adapted for multiple spin-echo sizes. In total, 34 different echo time (TE) values ranging from 10 to 1900 ms were used to capture the corresponding images. First, the *T*_2_ relaxation times were derived from a nonlinear least-squares fit of the average pixel values for multiple spin-echo measurements at each TE. Subsequently, the relaxation times (*T*_1_ and *T*_2_) were image-processed to generate a relaxation time map. Finally, the relaxivities (*r*_1_ and *r*_2_) were measured as the inverse of the relaxation time per mM.

### 3.4. Octanol−Water Partition Coefficients

We adopted a previously described method to measure the octanol−water partition coefficients [19]. Briefly, 1 mg of **Mn-NOTA-NP** was dissolved in 2 mL of water and 1-octanol (1:1 mixture). The vial containing the mixture was shaken for 30 s and placed on a rotator for gentle mixing to equilibrate for 48 h. After mixing, the sample was allowed to settle at room temperature for 24 h. Each layer of the Mn^2+^ concentration was determined using inductively coupled plasma-mass spectrometry (ICP-MS). The partition coefficients were calculated from the equation log P = log (C_o_/C_w_), where log P is the logarithm of the partition coefficient, and C_o_ and C_w_ are the concentrations of Mn in the 1-octanol and water layers, respectively.

### 3.5. Determination of Binding Constants

The binding affinity of the **Mn-NOTA-NP** chelate to human serum albumin (HSA) was determined using a previously published protocol [20]. A nonlinear increase in the proton paramagnetic relaxation rates for solutions containing 0.67 mM HSA at 64 MHz was fitted using the following equation:

$$\begin{array}{c} R_{1}^{p^{obs}}=1000\times \{\left(r_{1}^{f}s^{0}\right)+\frac{1}{2}(r_{1}^{c}\\ \;\;\;\;\;\;\;\;\;\;\;\;\;\;\;\;\;\;\;\;\;\;\;\;\;\;\;-r_{1}^{f})\left(\left(Np^{0}\right)+s^{0}+K_{a}^{-1}-\sqrt{{\left(\left(Np^{0}\right)+s^{0}+K_{a}^{-1}\right)}^{2}-4N\times s^{0}\times p^{0}},\}\right) \end{array}$$

where *N* is the number of independent interaction sites (*N* = 1), *K_a_* is the HSA binding constant, *p*^0^ is the HSA concentration, *s*^0^ is the complex paramagnetic concentration, and *r*_1_^c^ and *r*_1_^f^ are the relaxivities of the complex containing HSA and a free agent, respectively.

### 3.6. Transmetalation Kinetics

This experiment was performed using a previously reported method with slight modifications [35]. A total of 20 microliters of a 50 mM MES [2-(N-morpholino)ethanesulfonic acid] buffered solution (pH 6.0) of ZnCl_2_ was added to 1 mL of a buffered solution of the 1 mM Mn complex. The mixture was shaken for a while and immediately used to measure the solvent *T*_2_ as a function of time. Control studies were also conducted with Mn-EDTA, Gd-DTPA (Magnevist^®^), and Gd-DTPA-EOB (Primovist^®^) for comparison. The measurements were performed on a 3 T MRI scanner (Discovery MR750w 3.0 T, GE Healthcare, Chicago, IL, USA) at room temperature.

### 3.7. pH Stability

The pH-dependent changes in the longitudinal relaxation rate of **Mn-NOTA-NP** were evaluated on a scale ranging from pH 1 to 10. Samples of each pH buffer solution (pH 1, 4, 7, and 10) were brought from Daejung Chemicals (Siheung-si, Korea). The experiment was performed at room temperature for 72 h on a 3T system (128 MHz, Magnetom Tim Trio, Siemens, Korea Institute of Radiological and Medical Science). First, 950 µL of the buffer solution and 50 µL of the compound were added to a final volume of 1 mL. The pH was then adjusted using negligible volumes of diluted HCl/NaOH to decrease or increase the pH of the acidic and basic ranges while maintaining a constant complex concentration throughout the experiments. The imaging parameters for spin-echo images were as follows: TR = 2000; TE = 15; voxel size = 0.75 × 0.75 × 5.0 mm; 5.0 slice thickness; FOV read = 250 mm; FOV phase = 100.0%; scan time = 7 min 24 s.

### 3.8. Molecular Modeling Method

The atomic structure of MDR2 (also known as multidrug resistance-associated protein; MRP2) was accessed on September 1, 2022, and downloaded from the AlphaFold protein structure database in the pdb file format (https://alphafold.ebi.ac.uk/entry/Q92887). The atomic structures of OATP1B1 and OATP1B3 were predicted using the SWISS-MODEL homology modeling server (https://swissmodel.expasy.org/). The docking software used in this research were AutoDock MGLTools 1.5.7 (https://ccsb.scripps.edu/mgltools/, AutoDock Vina 1.1.2 (https://vina.scripps.edu/), RDKit (https://www.rdkit.org/), and Open-source PyMOL (https://github.com/schrodinger/pymol-open-source). Four ligands—**Mn-NOTA-NP**, Mn-EDTA-BTA, Mn-EDTA-EOB, and Gd-DTPA-EOB—were prepared, minimized in three-dimensional structures, and saved in the pdbqt file format by adding polar hydrogen and fixing charges. AutoDock Vina was primarily used for docking ligands into target proteins. The application searches for space boxes in the coordinate system of a specific area and predicts the binding ability of binding models. AutoDock Vina employed a grid space of 1.000 Å for all runs. The population size was set to 150 and the number of genetic algorithms to 27,000 in AutoDock Vina. The binding modes of the four ligands were generated and analyzed using open-source PyMOL.

### 3.9. Cell Culture

RAW 264.7 (mouse monocyte/macrophage cells, Catalog No. ATCC^®^CRL-2278) was obtained from ATCC (Manassas, VA, USA) and cultured in accordance with the manufacturer’s instructions. In addition, RAW 264.7 cells were cultured in RPMI-1640 medium obtained from Welgene (Daegu, Republic of Korea; Cat. LM011-01) containing 10% fetal bovine serum (FBS; Hyclone, Waltham, MA, USA; Catalog No. SH30919.03), and 1% penicillin-streptomycin solution (Gibco, Carlsbad, CA, USA; Catalog No. 11548876). Cells were cultured at 37 °C in a humidified incubator with a 5% CO_2_ atmosphere.

### 3.10. Cell Viability Assay

The cytotoxicity of **Mn-NOTA-NP** and Gd-DTPA in mouse macrophage cells (RAW 264.7) was investigated. RAW 264.7 cells were plated in 96-well plates containing 100 µL of complete media at 1 × 10^4^ cells/well. After 24 h, the medium was replaced with fresh medium containing varying concentrations of CAs (0, 0.5, 1, 5, 10, 25, 50, 100, and 200 µM), and the cells were incubated for another 22 h. The cells were then incubated for 2 h with CCK-8 solution (CCK-8, Dongin LS, Korea; Catalog No. CCK-3000). A microplate reader (SpectraMax i3, Molecular Devices, San Jose, CA, USA) was used to measure the absorbance at 450 nm.

### 3.11. Biodistribution

In total, 4 normal male Balb/c mice (7 weeks old, 20–25 g) were injected with 0.05 mmol/kg of **Mn-NOTA-NP** via the tail vein at various time points. The mice were anesthetized and sacrificed 15 min, 30 min, 1 h, and 24 h after injection. Mn levels were measured in the brain, heart, gallbladder, liver, kidneys, spleen, and intestine. Tissues were digested with 70% HNO_3_ and 30% H_2_O_2_ at 180 °C for 2 h. The Mn content was measured in the resulting clear diluted solution (3% nitric acid) using inductively coupled plasma optical emission spectroscopy (ICP-OES) (Optima 7300DV, Perkin Elmer, Waltham, MA, USA). The detection limit of this method was set to 0.010 ppm.

### 3.12. In Vivo MRI of Normal Mice

All animal experiments were approved and performed in accordance with the guidelines of the Kyungpook National University Animal Care Committee. The MRI study used 6-week-old male Balb/c mice weighing 20–25 g. Mice were anesthetized with 1.5% isoflurane in oxygen. Measurements were recorded before and after the injection of paramagnetic complexes via the tail vein. After each measurement, the mouse was woken from anesthesia and placed in a cage with unlimited food and water. During measurements, the animals were maintained at room temperature. A 3 T MRI scanner (Architect, GE Healthcare, USA) equipped with a homemade small-animal radio frequency (RF) coil was used to capture the MR images. The imaging parameters for spin-echo were as follows: for coronal images, TR/TE/flip angle = 450 ms/8.8 FKimms/111°, echo train length (ETL) = 3, matrix size = 256 × 192, FOV = 80 × 64 mm, thickness = 1.2 mm, number of acquisitions (NEX) = 4; for axial images, TR/TE/flip angle = 450 ms/7.2 ms/111°, ETL = 3, matrix size = 192 × 128, FOV = 80 × 64 mm, thickness = 1.5 mm, NEX = 2.

### 3.13. Liver Tumor Model and In Vivo MRI

All animal experiments using the liver tumor model were performed with the approval of the Korea Institute of Radiological and Medical Sciences. To prepare an orthotopic xenograft model, HepG2 cells were harvested and resuspended in serum-free medium containing 50% Matrigel, and 6-week-old nude mice (Balb/c, male) from DooYeol Biotech (Seoul, Republic of Korea) were anesthetized with 3% isoflurane. HepG2 cells and Matrigel were inoculated into the left hepatic lobe of the mice. In vivo imaging was performed on the mice two months after inoculation. **Mn-NOTA-NP** was prepared as 50 mM stock solutions and used at a dose of 0.05 mmol [Mn/kg]. MR images were obtained at 3.0 T (Magnetom Tim Trio, Siemens, Germany) using a homemade 6-channel rat body coil. The imaging parameters were as follows: *T*_1_-weighted 2D turbo spin echo (TSE) for coronal images, TR/TE/flip angle = 450 ms/10 ms/120°, ETL = 3, matrix size = 134 × 192, FOV = 60 mm, slice thickness = 1 mm, number of averages = 6; for axial images, TR/TE/flip angle = 450 ms/9.3 ms/120°, ETL = 3, matrix size = 134 × 192, FOV = 60 mm, slice thickness = 1.3 mm, number of averages = 4; *T*_2_-weighted TSE, TR/TE/flip angle = 1620 ms/37 ms/120°, matrix size = 256 × 256, FOV = 60 mm, slice thickness = 1 mm, number of averages = 4.

### 3.14. Image Analysis

On post-contrast MR images, anatomical positions with enhanced contrast were identified in the heart, liver, gallbladder, kidneys, and bladder. Additionally, signal intensities in specific regions of interest were measured quantitatively using Image J software (version 1.53e, National Institutes of Health, Bethesda, MD, USA). At each time point, the signal-to-noise ratio [SNR = P_signal_/P_noise_] and contrast-to-noise ratio [CNR = SNR_post_ − SNR_pre_] were calculated for the relevant tissues of each mouse.

### 3.15. Statistical Analysis

One-way analysis of variance (ANOVA) with the nonparametric Mann–Whitney U test was used to analyze the data. GraphPad PRISM (version 5.03, GraphPad PRISM software Inc., San Diego, CA, USA) and SPSS (version 25, SPSS Inc., Chicago, IL, USA) were used for the analyses. Additionally, a MATLAB curve-fitting tool was used to fit the curves. Data are presented as mean ± standard deviation (SD) or standard error of the mean (SEM) unless otherwise specified.

## 4. Conclusions

We developed a new macrocyclic Mn^2+^ complex comprising a π-conjugated naphthalene group and a NOTA-chelating ligand and evaluated its diagnostic potential as hepatobiliary MRI CAs. The in vivo biodistribution study and in vivo MRI investigation in mice suggested that **Mn-NOTA-NP** can promote excellent liver enhancement via a combination of hepatobiliary and renal clearance pathways, similar to that of Gd-DTPA-EOB. Moreover, in an HepG2 liver tumor model, **Mn-NOTA-NP** demonstrated enhanced tumor detection with paradoxically high tumor signal intensity. Additionally, ligand-binding simulations revealed that **Mn-NOTA-NP** binds to the transporters in a different manner than the other hepatobiliary agents investigated in this study. Hence, we believe that **Mn-NOTA-NP** can be an excellent alternative to Gd^3+^-based hepatobiliary agents because it exhibits optimal properties as a liver-specific MRI agent.

## Figures and Tables

**Table 2:** Docking scores for **Mn-NOTA-NP**, Mn-EDTA-BTA, Mn-EDTA-EOB, and Gd-DTPA-EOB with organic anion-transporting polypeptides (OATPs) and multidrug resistance-associated protein 2 (MRP2). Data expressed as kcal/mol.

Compounds	OATP1B1	OATP1B3	MRP2
**Mn-NOTA-NP**	−9.281	−5.532	−4.579
Gd-DTPA-EOB ^c^	−13.25	−11.54	−3.976
Mn-EDTA-BTA	−7.392	−10.172	−5.731
Mn-EDTA-EOB ^c^	−9.36	−11.58	−5.149

^c^ Data collected from the cited reference [19].

## Data Availability

Not applicable.

## Supplementary Materials

The following supporting information can be downloaded at: https://www.mdpi.com/article/10.3390/ph16040602/s1, Figure S1: ^1^H NMR spectrum of compound **2**; Figure S2: ^13^C NMR spectrum of compound **2**; Figure S3: High-resolution mass spectrum of compound **2**; Figure S4: ^1^H NMR spectrum of compound **3**; Figure S5: High-resolution mass spectrum of compound **3**; Figure S6: ^1^H NMR spectrum of compound **NOTA-NP**; Figure S7: ^13^C NMR spectrum of compound **NOTA-NP**; Figure S8: High-resolution mass spectrum of compound **NOTA-NP**; Figure S9: HR-ESI-MS spectrum of compound **Mn-NOTA-NP**; Figure S10: HPLC spectrum of compound **Mn-NOTA-NP**; Figure S11: HSA binding of **Mn-NOTA-NP**; Figure S12: pH stability of **Mn-NOTA-NP**; Figure S13: Schematic representations of variable interactions between Mn-EDTA-BTA and (a) OATP1B1, (b) OATP1B3, and (c) MRP2 transporters; Figure S14: Schematic representations of variable interactions between (a) Mn-EDTA-EOB and (b) Gd-DTPA-EOB with MRP2 transporter.
