# Supplementary material for: Manganese (II) Complex of 1,4,7-Triazacyclononane-1,4,7-Triacetic Acid (NOTA) as a Hepatobiliary MRI Contrast Agent
Source: Pharmaceuticals (Basel). 2023 Apr 17;16(4):602. doi: 10.3390/ph16040602 (PMC10141232; doi:10.3390/ph16040602)
Supplement: Supplementary file 1 [file pharmaceuticals-16-00602-s001.zip › pharmaceuticals-2295052-supplementary.pdf]

Supplementary Materials

Manganese (II) Complex of 1,4,7-Triazacyclononane-1,4,7-Triacetic Acid (NOTA) as a Hepatobiliary MRI Contrast Agent

Md. Kamrul Islam [1], Ah-Rum Baek [1], Byeong-Woo Yang [2], Soyeon Kim [3], Dong Wook Hwang [4], Sung-Wook Nam [4], Gang-Ho Lee [5] and Yongmin Chang [2,4,6,*]

1 Institute of Biomedical Engineering Research, Kyungpook National University, Daegu 41405, Republic of Korea

2 Department of Medical and Biological Engineering, School of Medicine, Kyungpook National University, Daegu 41944, Republic of Korea

3 Division of RI-Convergence Research, Korea Institute of Radiological and Medical Sciences, Seoul 01812, Republic of Korea

4 Department of Molecular Medicine, School of Medicine, Kyungpook National University, Daegu 41944, Republic of Korea

5 Department of Chemistry, Kyungpook National University, 80, Daehak-ro, Buk-gu, Daegu 41566, Republic of Korea

6 Department of Radiology, Kyungpook National University Hospital, Daegu 41944, Republic of Korea

* Correspondence: ychang@knu.ac.kr; Tel.: +82-53-420-5471

Table of Contents

1.	**Figure S1.** ^{1}H NMR spectrum of compound **2**.	S3
2.	**Figure S2.** ^{13}C NMR spectrum of compound **2**.	S3
3.	**Figure S3.** High-resolution mass spectrum of compound **2**.	S4
4.	**Figure S4.** ^{1}H NMR spectrum of compound **3**.	S4
5.	**Figure S5.** High-resolution mass spectrum of compound **3**.	S5
6.	**Figure S6.** ^{1}H NMR spectrum of compound **NOTA-NP**.	S5
7.	**Figure S7.** ^{13}C NMR spectrum of compound **NOTA-NP**.	S6
8.	**Figure S8.** High-resolution mass spectrum of compound **NOTA-NP**.	S6
9.	**Figure S9.** HR-ESI-MS spectrum of compound **Mn-NOTA-NP**.	S7
10.	**Figure S10.** HPLC spectrum of compound **Mn-NOTA-NP**.	S7
11.	**Figure S11.** HSA binding of **Mn-NOTA-NP**.	S8
12.	**Figure S12.** pH Stability of **Mn-NOTA-NP**.	S8
13.	**Figure S13.** Schematic representations of variable interactions between Mn-EDTA-BTA and (a) OATP1B1, (b) OATP1B3, and (c) MRP2 transporters.	S9
14.	**Figure S14.** Schematic representations of variable interactions between (a) Mn-EDTA-EOB and (b) Gd-DTPA-EOB with MRP2 transporter.	S9

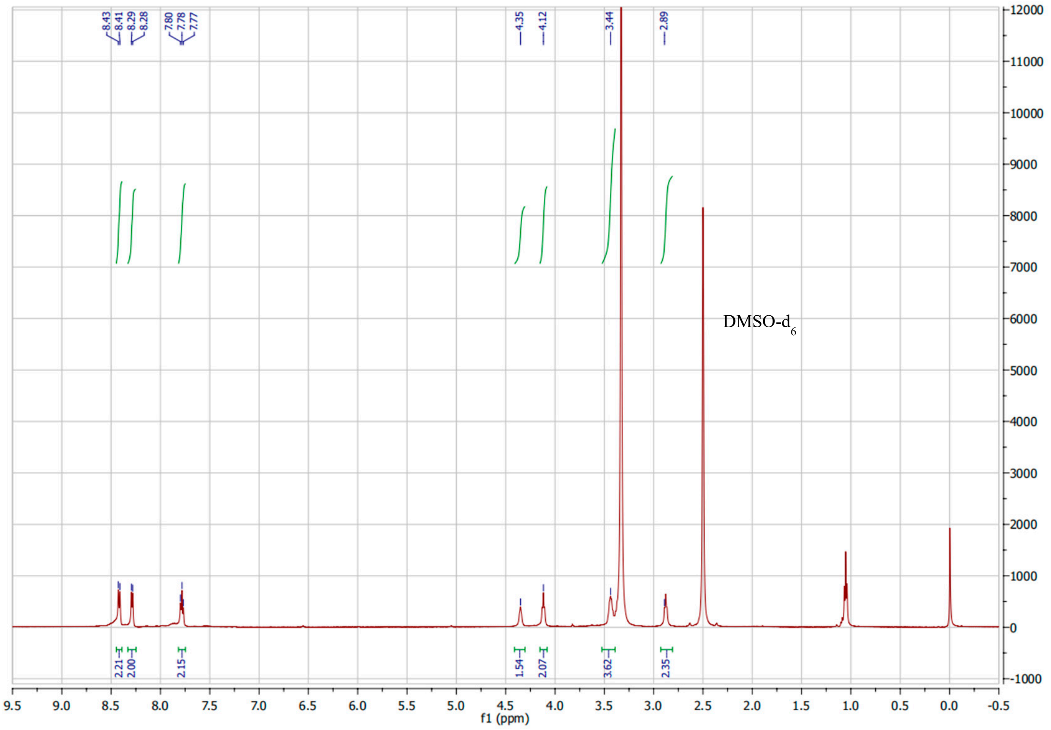

Figure S1. ^{1}H NMR spectrum of compound **2**.

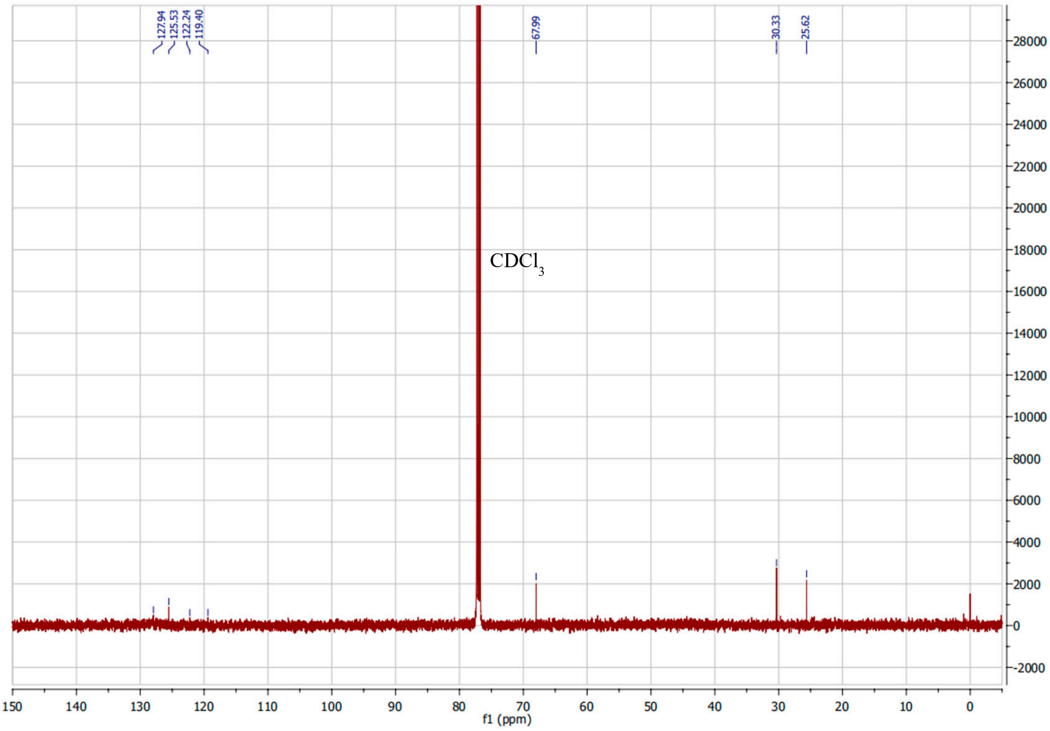

Figure S2. ^{13}C NMR spectrum of compound **2**.

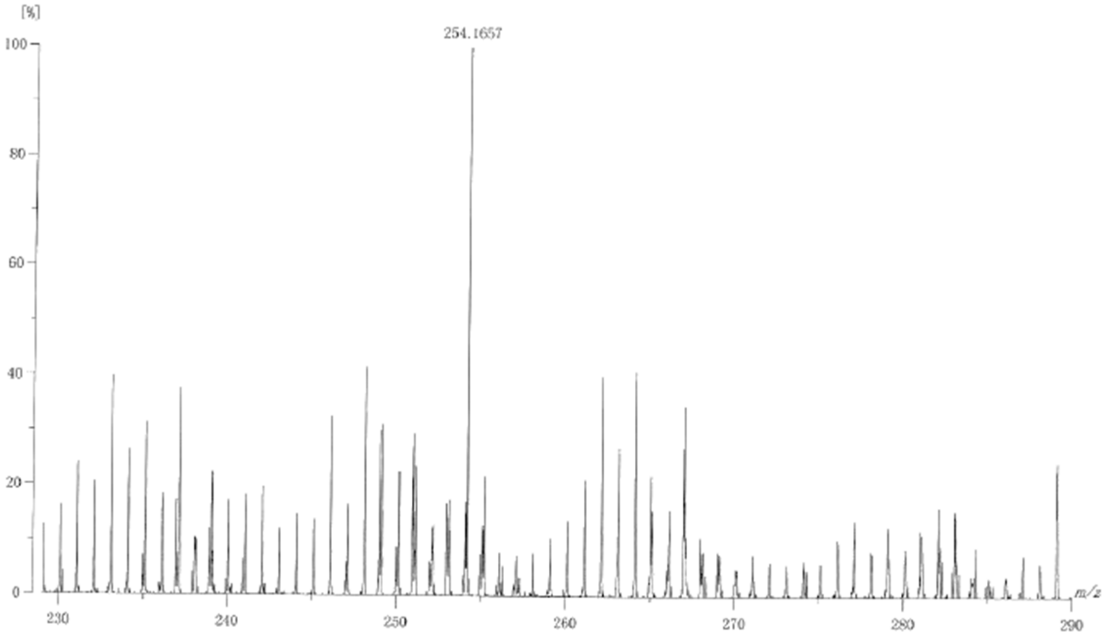

Figure S3. HR-FAB-MS spectrum for compound **2**.

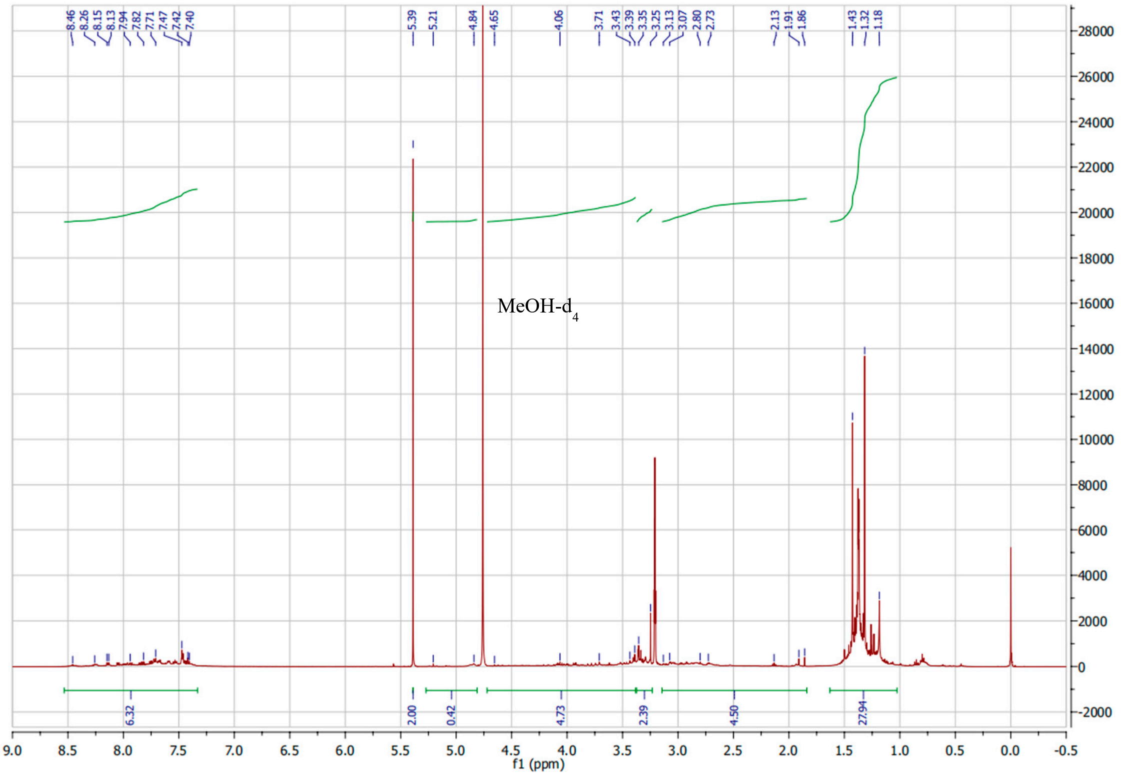

Figure S4. ^{1}H NMR spectrum of compound **3**.

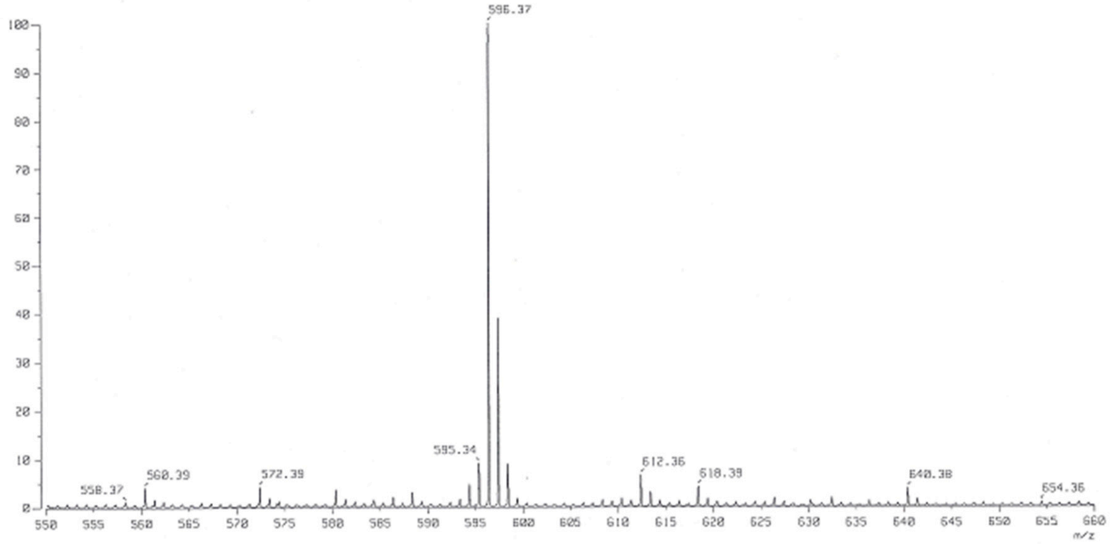

Figure S5. HR-FAB-MS spectrum for compound **3**.

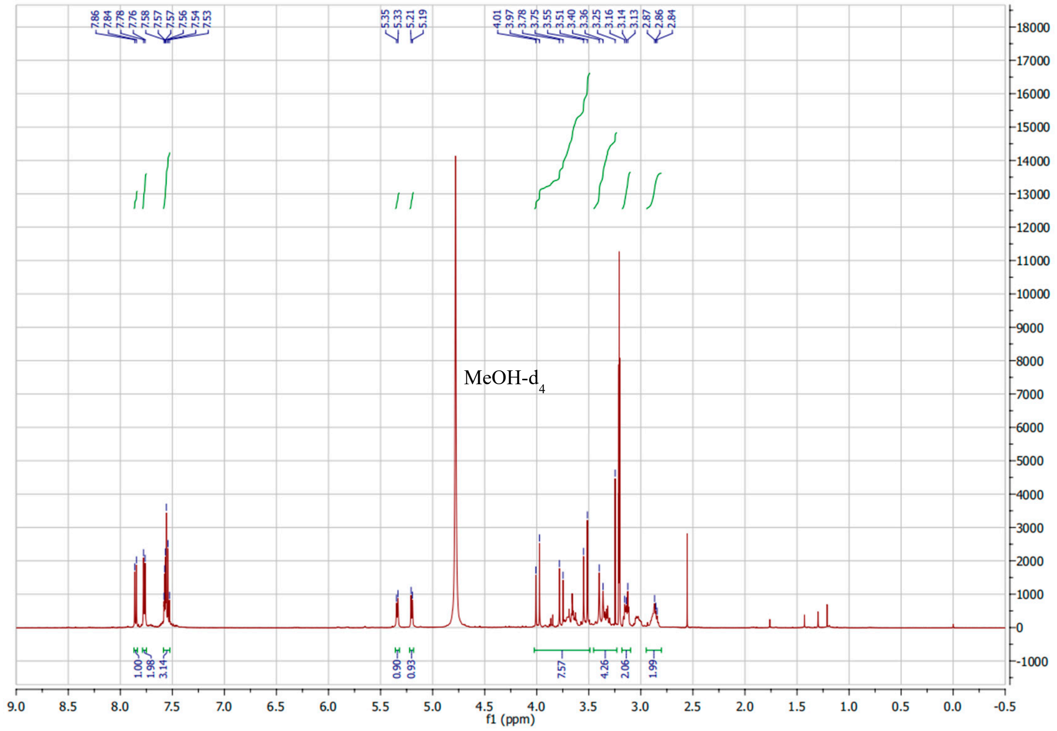

Figure S6. ^{1}H NMR spectrum of **NOTA-NP**.

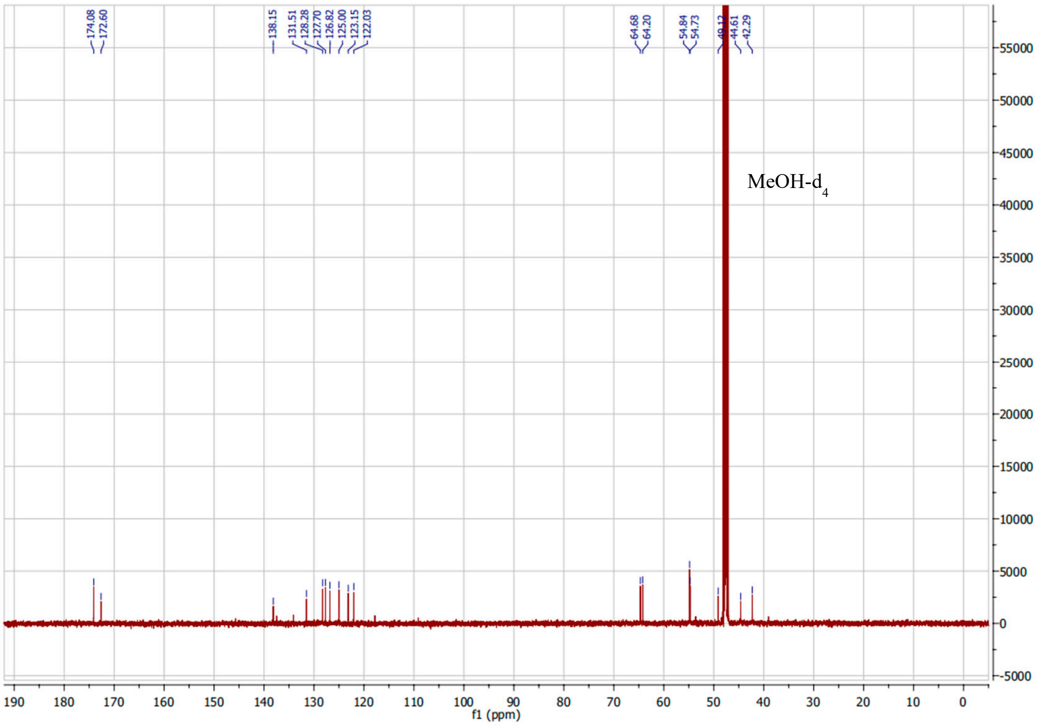

Figure S7. ^{13}C NMR spectrum of **NOTA-NP**.

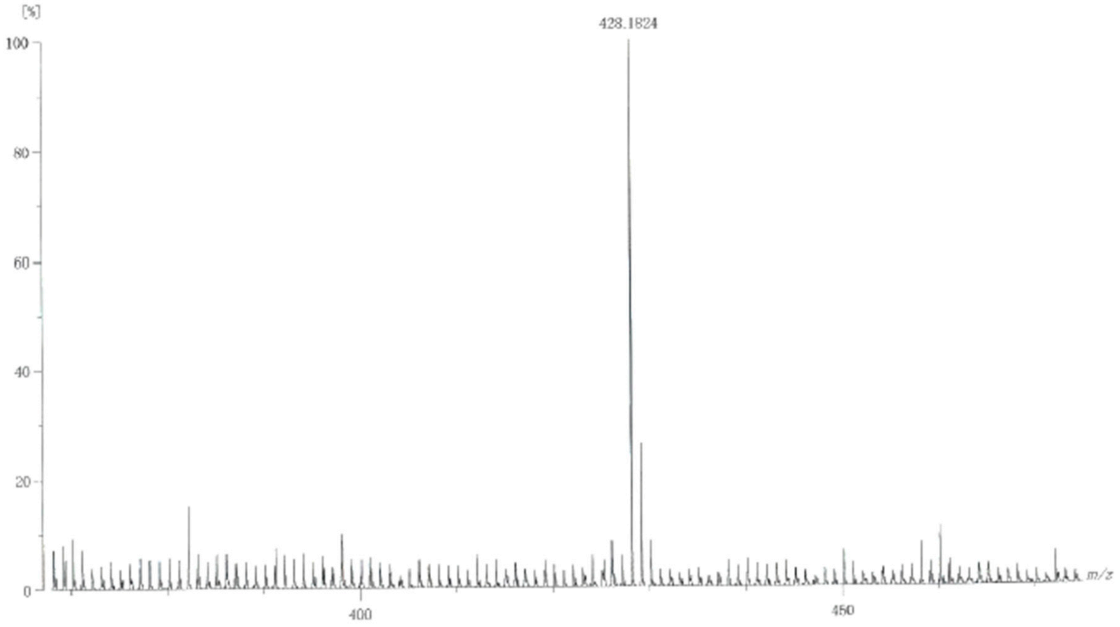

Figure S8. HR-FAB-MS spectrum of compound **NOTA-NP**.

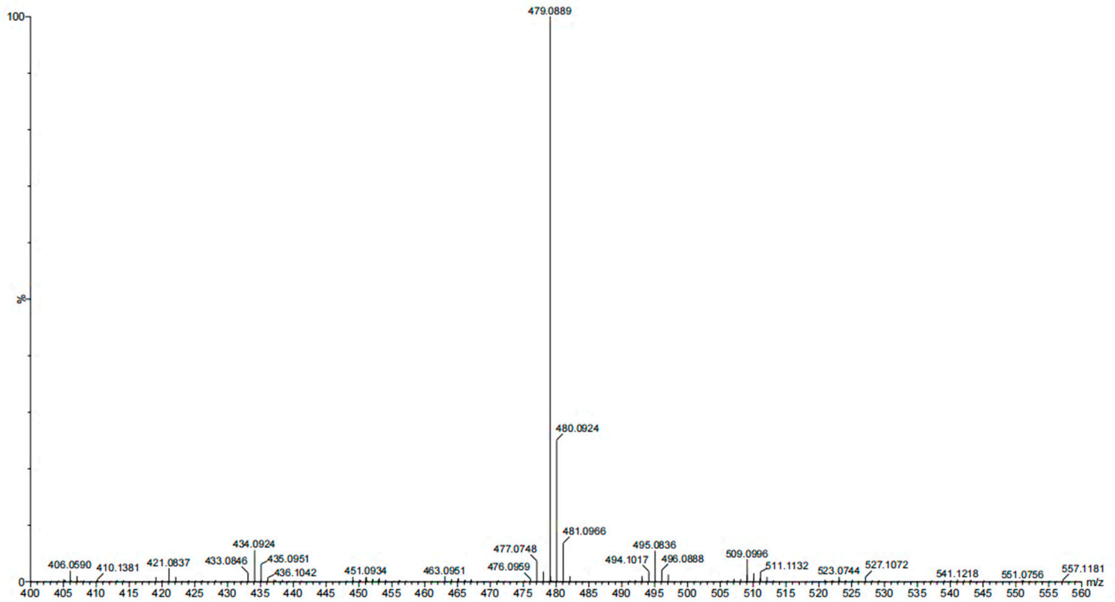

Figure S9. HR-ESI-MS spectrum of compound **Mn-NOTA-NP**.

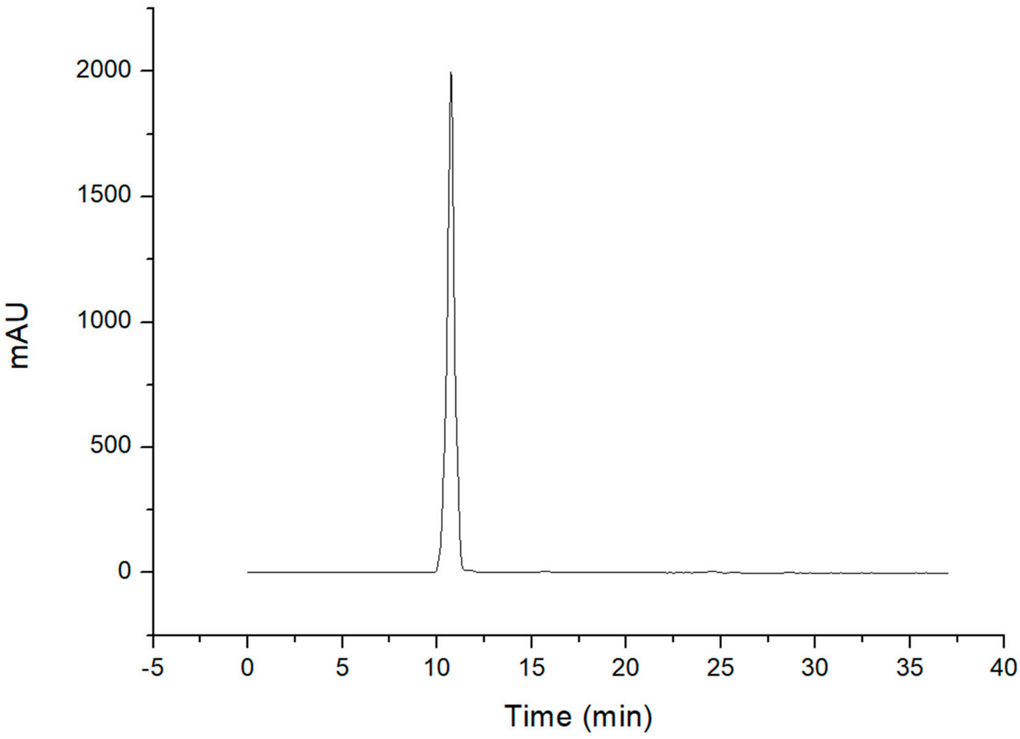

Figure S10. HPLC spectrum of compound **Mn-NOTA-NP** at 254 nm detection with 96.05% purity.

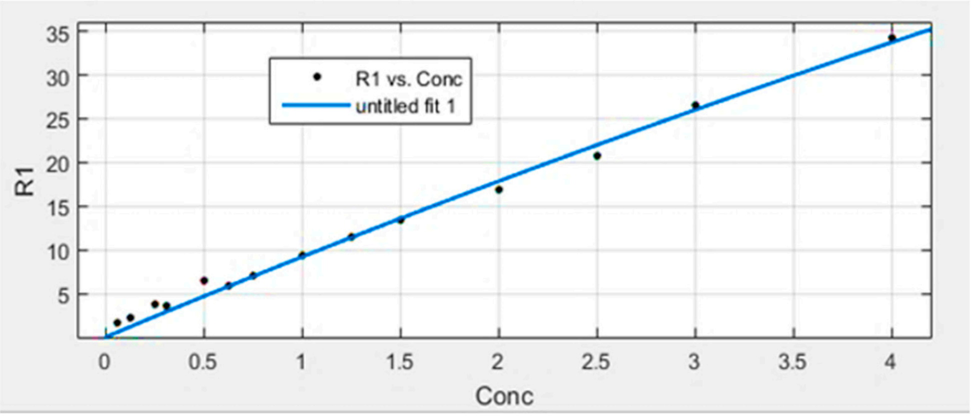

Figure S11. Proton longitudinal paramagnetic relaxation rates of **Mn-NOTA-NP** as a function of Mn concentration in an aqueous solution of HSA (0.67 mM) at 64 MHz and 293 K.

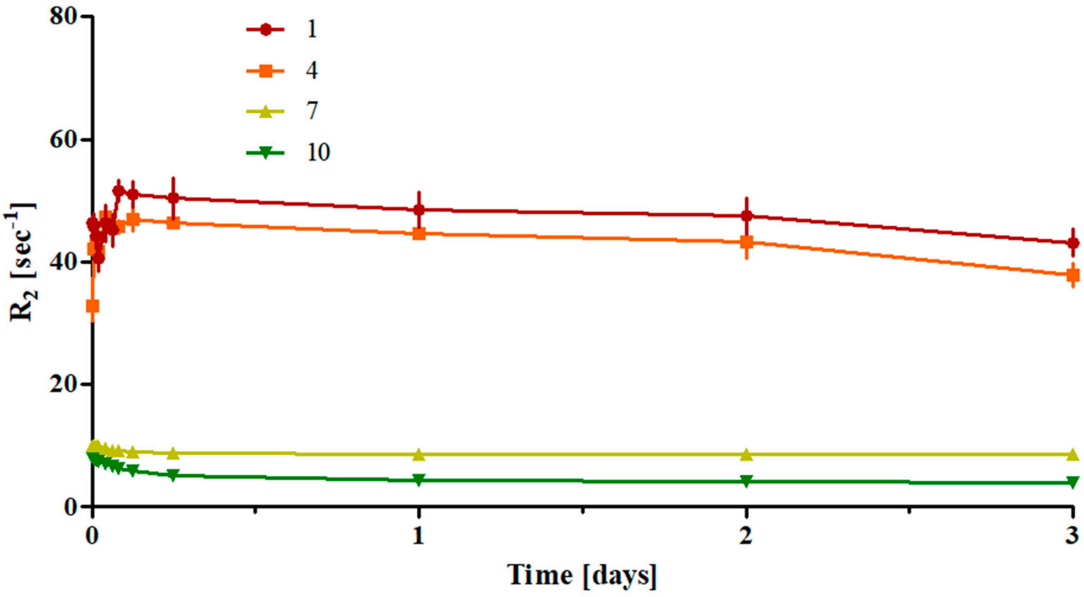

Figure S12. Stability of **Mn-NOTA-NP** ([Mn] = 1.0 mM) in different pH buffer solutions as a function of incubation time at 3 T and 293 K.

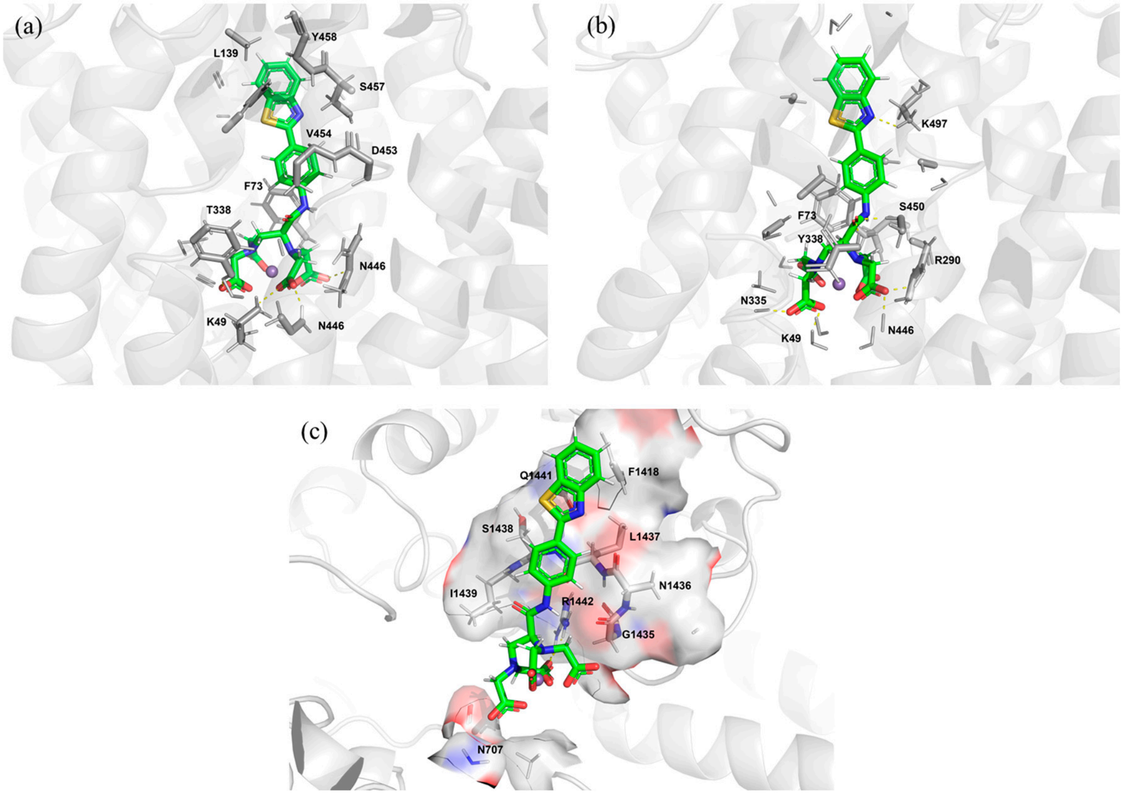

Figure S13. Schematic representations of variable interactions between Mn-EDTA-BTA and (a) OATP1B1, (b) OATP1B3, and (c) MRP2 transporters. The yellow line represents hydrogen bonds.

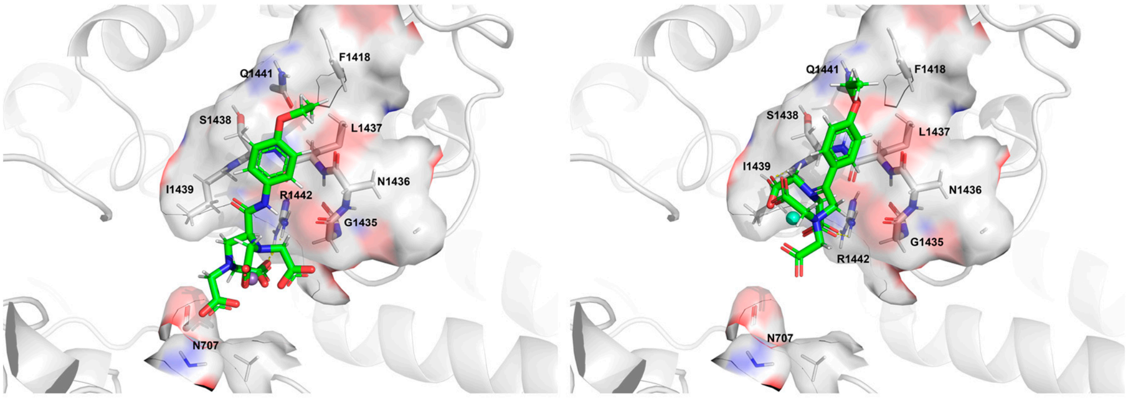

(a) (b)

Figure S14. Schematic representations of variable interactions between (a) Mn-EDTA-EOB and (b) Gd-DTPA-EOB with MRP2 transporter. The yellow line represents hydrogen bonds.
